# Enzyme-Assisted Extraction to Obtain Phenolic-Enriched Wine Lees with Enhanced Bioactivity in Hypertensive Rats

**DOI:** 10.3390/antiox10040517

**Published:** 2021-03-26

**Authors:** Raúl López-Fernández-Sobrino, Maria Margalef, Cristina Torres-Fuentes, Javier Ávila-Román, Gerard Aragonès, Begoña Muguerza, Francisca Isabel Bravo

**Affiliations:** 1 Nutrigenomics Research Group, Department of Biochemistry and Biotechnology, Universitat Rovira i Virgili, 43007 Tarragona, Spain; raul.lopez@urv.cat (R.L.-F.-S.); maria.margalef.jornet@gmail.com (M.M.); cristina.torres@urv.cat (C.T.-F.); franciscojavier.avila@urv.cat (J.Á.-R.); gerard.aragones@urv.cat (G.A.); franciscaisabel.bravo@urv.cat (F.I.B.); 2 Technological Center of Nutrition and Health (CTNS), TECNIO, CEICS, 43204 Reus, Spain

**Keywords:** hypertension, phenolic compounds, hydrolysate, spontaneously hypertensive rats, bioactive compounds, enzymatic hydrolysis, UHPLC, grape by-products

## Abstract

The antihypertensive effect of the soluble fraction of wine lees (WL) from Cabernet variety grapes was recently reported by our group. This blood pressure (BP)-lowering effect was attributed to the presence of flavanols and anthocyanins. In this context, phenolic-enriched wine lees (PWL) could potentially exhibit a stronger bioactivity. Therefore, the aim of this study was to obtain a soluble fraction of WL with increased phenolic content and evaluate its functionality. The PWL were obtained using an enzyme-assisted extraction based on the hydrolysis of WL proteins with Flavourzyme^®^. They contained 57.20% more total phenolic compounds than WL, with anthocyanins and flavanols being the largest families present. In addition, PWL also showed greater angiotensin-converting enzyme inhibitory and antioxidant activities. Finally, the antihypertensive activity of the PWL was evaluated in spontaneously hypertensive rats. A single dose of 5 mL/kg body weight of PWL showed a greater BP-lowering effect than the one shown by WL. Moreover, this antihypertensive effect was more prolonged than the one produced by the antihypertensive drug Captopril. These results demonstrate that enzymatic protein hydrolysis is a useful method to maximize the extraction of phenolic compounds from WL and to obtain extracts with enhanced functionalities.

## 1. Introduction

Nowadays, it is known that some dietary components play an important role in the prevention of hypertension (HTN) [1]. Thus, an increase of fruit and vegetable intake has been evidenced to reduce the risk of cardiovascular disease (CVD) in hypertensive subjects [2,3]. Their health benefits are attributed to the presence and synergetic effect of different bioactive compounds including fiber, vitamins or phenolic compounds [4]. In the last two decades, phenolic compounds have been widely studied for their beneficial health effects, including antihypertensive activity [5,6,7].

There are different mechanisms related to the blood pressure (BP)-lowering effect of phenolic compounds. Some of these have been referred to their antioxidant capacity [6,8]. In fact, it has been reported for anthocyanin, flavanol or flavonol rich extracts that the oxidative stress improvement is one of the mechanisms responsible for their antihypertensive properties [9,10,11]. In addition, phenolic compounds can also act on HTN via the inhibition of angiotensin-converting enzyme (ACE) [12]. This enzyme is key in the BP regulation produced by the renin-angiotensin-aldosterone system (RAAS). In fact, ACE inhibition is usually used in HTN treatment [13,14]. Although synthetic ACE inhibitors such as Enalapril or Captopril are very efficient in reducing BP, in some cases they can cause unwanted side effects [15,16]. Thus, new antihypertensive compounds, acting on different HTN targets, are being highly investigated, mainly from natural sources. In this regard, the ACE inhibitory (ACEi) activity of different phenolic compounds has been demonstrated in in vitro studies [17]. Phenolics do not reach the potency of drugs commonly used in the treatment of HTN as Captopril but they may be considered as naturally functional compounds since their dietary intake can be as high as 1 g/day [17].

Agri-food by-products have recently emerged as a new source of bioactive compounds [18]. In this regard, winery by-products (seeds, skin, pomace, stems, or lees) have been used to obtain extracts rich in phenolic compounds with antioxidant and/or antihypertensive properties [10,11,19,20,21]. However, these phenolic compounds can be in the food matrix in free form or covalently bound to soluble or insoluble substances [22]. For example, tannins, phenolic compounds present in winery by-products, form complexes with proteins [23]. Thus, extraction methods must be used to release these bioactive compounds [22]. However, some of these methods produce residual substances and more eco-friendly alternatives are being developed. In this regard, enzyme-assisted extraction has emerged as a green technique that brakes or softens the cell wall to release bioactive compounds [24]. Agri-food by-products contain macromolecules that can be susceptible to hydrolysis such as proteins, cellulose, lignin or hemicellulose [25]. In fact, this reaction has already been used to release phenolic compounds from the cell wall matrix [22]. Specifically, protein hydrolysis has been evidenced to be a good method to release proteins and phenolic compounds [26,27].

Wine lees (WL), a winery by-product, account for 25% of the waste from the winemaking process [28]. The Council Regulation (EEC) No. 337/79 defined this by-product as “the residue that forms at the bottom of recipients containing wine, after fermentation, during storage or after authorized treatments, as well as the residue obtained following the filtration or centrifugation of this product” [29]. WL can be classified according to the stage of the wine making process, as first-fermentation WL, second-fermentation WL, and aging WL (after wine alcoholic fermentation, malolactic fermentation or aging in wood barrels, respectively) [30]. WL can be separated into two phases after centrifugation or filtration of WL, the solid and the liquid phases. The proportion of both phases can vary depending on the type of WL or the procedure to obtain the WL, since water can be added to remove WL from wine tanks. The solid phase is mainly composed of yeasts, insoluble carbohydrates, phenolic compounds, proteins, lignin, tartrates, and other materials such as grape skins [31]. Regarding the soluble phase, this is composed of organic acids, ethanol, and soluble phenolic compounds [19,31]. A previous study from our group evidenced the antihypertensive effect of the soluble fraction of WL from grapes of the Cabernet variety [19]. Phenolic analysis revealed that this fraction was rich in flavanols and anthocyanins. However, since only a centrifugation was performed and no particular extraction method was applied, phenolic compounds from the non-soluble fraction of WL were not extracted. In this regard, the presence of phenolic compounds in the solid fraction, bound to yeast cell walls, has been reported [32]. Enzymatic-assisted extraction using Glucanex^®^ and Mannaway^®^ (β-1, 3 glucanase and β-1,4-mannanase activities, respectively) has been efficiently used in WL as treatment previous to a solid–liquid extraction to release anthocyanins or glycocompounds from the non-soluble fraction [33,34]. Considering this evidence, the aim of this study was to increase the release of WL phenolic compounds by protein hydrolysis of WL to maximize the phenolic yield, improving the valorization of this by-product. The antioxidant, ACEi and antihypertensive activities of the phenolic-enriched WL (PWL) were also evaluated.

## 2. Materials and Methods

### 2.1. Chemicals and Reagents

Flavourzyme^®^ 1000 L (EC 3.4.11.1, 500 leucine amino peptidase units (LAPU)/g from *Aspergillus oryzae*) was kindly provided by Novozymes (Bagsværd, Denmark). ACE (EC 3.4.15.1), 2,2-diphenyl-1-picryl-hydrazyl-hydrate (DPPH), picrylsulfonic acid and acetonitrile for HPLC were purchased from Sigma-Aldrich (Madrid, Spain). Captopril (PubChem CID: 44093) and resveratrol were provided by Santa Cruz Biotechnology (Dallas, TX, USA) and Carl Roth (Karlsruhe, Germany), respectively. Folin–Ciocalteu reagent, coumaric acid, quercetin, gallic acid, catechin, and epicatechin were purchased from Fluka/Sigma-Aldrich. 4-Hydroxybenzoic acid, caffeic acid, procyanidin dimer B2, vanillic acid, and malvidin-3-*O*-glucoside were purchased from Extrasynthése (Lyon, France). Ferulic acid, cyanidin-3-*O*-rutinoside, and peonidin-3-*O*-rutinoside were purchased from PhytoLab (Vestenbergsgreuth, Germany). Analytical grade reagents were always used.

### 2.2. Preparation of the Wine Lees Hydrolysate

WL were kindly provided by the cellar Grandes Vinos y Viñedos S.A (Cariñena PDO area, Cariñena, Spain). They were collected after racking the red wine (first-fermentation WL), which was made from grapes of Cabernet variety. The preparation of the new PWL was as follows: 10 mL of WL were mixed with the commercial enzyme solution Flavourzyme^®^ (enzyme/substrate ratio, 80 LAPU/g protein). Hydrolysis was carried out at 25 °C for 2 h at pH 4.0 and 250 rpm in a MaxQ Orbital Shaker Thermo Scientific (Thermo Fisher Scientific, Waltham, MA, USA). These enzymatic conditions were set according to a previous study focus on selecting the most appropriate enzymatic preparation and choosing process conditions easily applicable on an industrial scale (data not shown). Reaction was finished adding 1 M HCl to decrease the solution pH to 3. The final volume of the hydrolysate was 11.25 mL. Subsequently, the hydrolysate was centrifuged at 3000× *g* for 15 min at 4 °C to eliminate non-soluble particles and the supernatant (PWL) was collected for analysis. In addition, no-hydrolysis control WL (0 WL) was also subjected to the same procedure but replacing the enzyme volume by Milli-Q water.

### 2.3. Characterization of Wine Lees

Initial WL consisted of 28.0 ± 6.6% (*w*/*v*) solid, determined by AOAC official method [35], 27.14 ± 1.13% (*w*/*w*), protein, determined applying the factor 6.25 to the total nitrogen content measured by the Kjeldahl method [35] and 100.50 ± 1.36 mg GA/g total phenolic compound contents, determined by the Folin–Ciocalteu method according to Iglesias-Carres et al. [36]. Gallic acid (GA) was used as the calibration curve (40–400 mg/L) and mg GA equivalents per mL of WL (mg GAE/mL) were used to express the results. Protein and total polyphenol contents were expressed per gram of dry weight. The analysis was done in triplicate.

The percentage of solid (%, *w*/*v*) and total protein contents (%, *w*/*w*) of both 0 WL and PWL were determined according the AOAC official methods [35] and total phenolic compounds (mg GAE/g of dry weight), were determined by the Folin–Ciocalteu method [36], as already mentioned. The determination of the total amino acid content was carried out following the method described by Mas-Capdevila et al. [37]. Briefly, proteins in the samples suffered both acidic and basic hydrolysis under heat conditions for 1.5 h using increasing temperatures up to 150 °C. Identification and quantification of the amino acids in the samples were performed using high-performance liquid chromatography coupled to electrospray ionization and quadrupole time-of-flight mass spectrometry (UHPLC-Q-TOF/MS). All analyses were done at least in duplicate.

The hydrolysis degree of the PWL was determined by the TNBS (2,4,6-trinitrobenzenesulfonic acid) method according to Aldler–Nissen through the determination of free α-amino acid groups [38]. Total hydrolysis was performed adding 6 N HCl to the sample, incubating it for 24 h at 110 °C. Leucine was used as a calibration curve. The protein content used to calculate the hydrolysis degree was the one obtained by the Kjeldahl method. The analysis was done in triplicate.

All the analyses were carried out using the samples directly without any extraction method.

### 2.4. Identification and Quantification of the Phenolic Profile

The phenolic profiles of 0 WL and PWL were also analyzed. Separation, identification, and quantification of anthocyanin and non-anthocyanin phenolic compounds were performed following the method described by López–Fernández–Sobrino et al. [19]. 0 WL and PWL were diluted with water:methanol (50:50, *v*:*v*) and directly injected in a 1290 UHPLC Infinity II series coupled to a Q-TOF/MS 6550 (Agilent Technologies, Palo Alto, CA, USA). Both positive and negative ionization ([M-H]– or [M-H]+) were used to identify parental ions and fragmentation patterns as shown by López–Fernández–Sobrino et al. [19]. Commercial standards were used to construct a calibration curve to carry out the quantitative analysis of coumaric acid, procyanidin dimer B2, quercetin, catechin, epicatechin, 4-hydroxybenzoic acid, gallic acid, malvidin-3-*O*-glucoside, caffeic acid, vanillic acid, ferulic acid, cyanidin-3-*O*-rutinoside, peonidin-3-*O*-rutinoside, and resveratrol. For the rest of the compounds, the analysis was semi-quantitative using the calibration curve of the commercial standard having the most similar structure to the analyzed compound.

### 2.5. Antioxidant Activity

DPPH assay was used to determine the antioxidant activity of both samples following the method described by Shen et al. 2010 [39] with some modifications. An aliquot of 500 μL of sample was mixed with 200 μL of DPPH 0.5 mM (in ethanol). After vortexing, the samples were kept in the dark at room temperature for 30 min. Absorbance was measured at 517 nm. Ascorbic acid was used as a positive control, and samples were diluted in ethanol at different concentrations (0–400 μg/mL). A non-lineal fit was performed on the experimental data to calculate the EC_50_, that is the quantity necessary to reduce 50% of radical scavenging. DPPH radical scavenging activity was expressed as percentage of activity (%) or EC_50_ (µg of dry weight/mL of dissolution). Data are presented as the mean value of three determinations ± SD.

### 2.6. ACE Inhibitory Activity

ACEi activity was determined according to López–Fernández–Sobrino et al. [19]. The method uses the fluorescence compound *o*-Abz-Gly-*p*-Phe(NO_2_)-Pro-OH as ACE substrate. In the presence of an ACE inhibitor, a partial or total loss of fluorescence is produced during the reaction depending on the ACE inhibitory potential of the studied compound. Specifically, in this study, fluorescence was measured at 30 min (37 °C), using 360 nm and 400 nm as excitation and emission wavelengths respectively. ACEi activity was expressed as percentage of inhibition (%) or IC_50_ (µg of dry weight/mL of dissolution). The comparison of the percentage of ACEi activity of both WL was carried out using the same volume of the samples, 0.16 µL. IC_50_ was calculated by constructing a dose-response curve. A linear approximation regression was used. Data are presented as the mean value of three determinations ± SD. Captopril, a synthetic ACE inhibitor, was used to validate the methodology.

### 2.7. Antihypertensive Effect

Male spontaneously hypertensive rats (SHR) (17–20 weeks old, weighing 350–400 g) were purchased from Charles River Laboratories España S.A. (Barcelona, Spain). Rats were singly housed in a temperature-controlled animal quarter (22 °C) with a 12 h light/dark period. During the experiment, they had free access to standard chow (Panlab A04, Panlab, Barcelona, Spain) and to water.

After a 10-day adaptation period and two weeks of a training period, animals were given a single dose of the different tested compounds between 9 and 10 a.m. by oral gavage. A dose of 5 mL/kg body weight (bw) of PWL and 0 WL was administered to SHR. Captopril (50 mg/kg bw, a known antihypertensive drug) and tap water were used as controls (positive and negative, respectively).

BP was measured in the SHR before and after (2, 4, 6, 8, 24, 48, and 72 h) administration of treatments, using the tail-cuff method and the LE 5002 system (Letica, Hospitalet, Barcelona, Spain) according to Quiñones et al. [40]. Prior to BP measurements and to facilitate the detection of the tail artery pulsations, SHR were kept at 38 °C for 10 min. Changes in systolic and diastolic BP (SBP and DBP, respectively) were expressed as the differences in these variables before and after the administration for each treatment. Data are shown as the mean values ± standard error of mean (SEM) for a minimum of six experiments. BP measurements were recorded by the same person in a peaceful environment to minimize stress-induced variations.

Experimental in vivo studies were carried out following the European Communities Council Directive (86/609/EEC). In addition, the protocol was reviewed and approved by the Animal Ethics Review Committee for Animal Experimentation of the Universitat Rovira i Virgili and further approved by Generalitat de Catalunya (permission number 10780).

### 2.8. Statistical Analysis

Differences between 0 WL and the PWL in their amino acid content, total phenolic content, phenolic profile, antioxidant activity, and ACEi activity were analyzed by Student’s T-test. Two-way analysis of variance (two-way ANOVA) and Tukey test as post hoc were used to detect differences between treatments in BP over the evaluated time. One-way ANOVA was used to analyze the BP treatment differences at a specific time point. Differences between compounds were considered significant when *p* < 0.05. GraphPad Prism 7.04 for Windows (GraphPad Software, San Diego, CA, USA) was used to perform the statistical analysis. Grubbs’ test was used to detect outliers.

## 3. Results

### 3.1. Wine Lees Composition

Samples were characterized according to their humidity, total protein, total amino acid, total phenolic content, and hydrolysis degree. Solid contents of 0 WL and PWL were 2.49 ± 0.05% and 2.78 ± 0.16%, respectively. Total protein contents of the 0 WL and PWL were 26.08 ± 0.60 and 27.50 ± 0.71%, respectively. Total amino acid compositions of the 0 WL and PWL are shown in Table 1. The amino acid content of the 0 WL was 24.83 mg/g of dry product, with Pro being the major amino acid found. Regarding PWL, total amino acid concentration was 1.7 times higher (41.20 mg/g of dry product) than the one shown by the WL without hydrolysis. An increase in the content of Pro, Leu, Ile and Val (1.4, 6.6, 5.6 and 5.5 times higher, respectively) was observed in the hydrolysate compared to the 0 WL. In addition, PWL also contained Trp, while this amino acid residue was not found in the 0 WL. The hydrolysis degree of the PWL was 7.61 ± 0.65%.

Total phenolic content of PWL, measured by the Folin–Ciocalteu method, was 160.06 ± 0.32 mg GAE/g, significantly higher than the one observed by 0 WL (148.03 ± 0.48 mg GAE/g).

### 3.2. Determination of the Phenolic Profile in Wine Lees Samples

**Figure 1:**
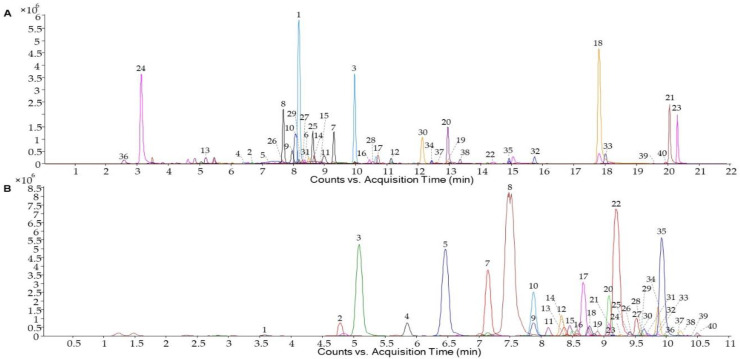
Phenolic profile of wine lees hydrolysate. Extracted ion chromatograms (EIC) of non-anthocyanin (**A**) and anthocyanin (**B**) phenolic compounds analyzed by UHPLC-(ESI-)-Q-TOF-MS and UHPLC-(ESI+)-Q-TOF-MS, respectively. Identified individual compounds are numbered according to Table 2 and Table 3.

**Figure 2:**
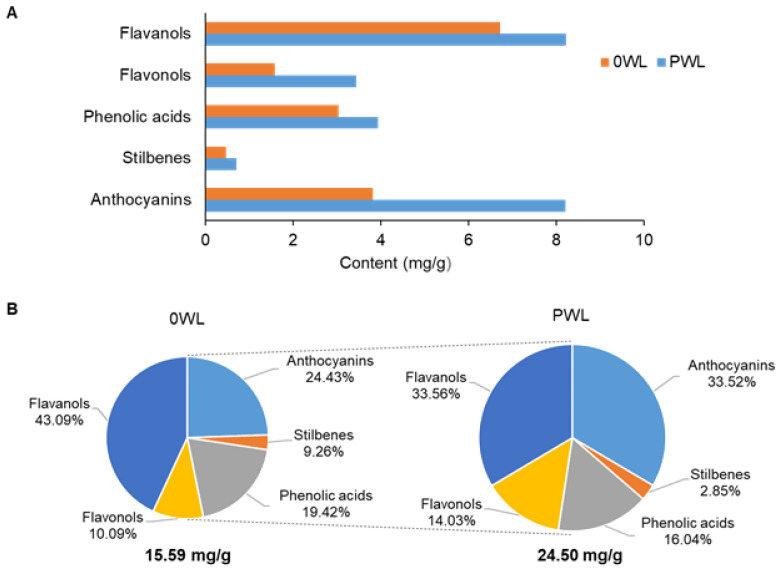
Flavanols, flavonols, phenolic acids, stilbenes, and anthocyanins content analyzed by UHPLC-(ESI)-Q-TOF-MS of control wine lees (0 WL) and phenolic-enriched wine lees (PWL) (**A**). Total phenolic compounds and percentages of the different families of phenolic compounds in 0 WL and PWL (**B**).

Phenolic profiles of the 0 WL and PWL were determined. As example, Figure 1 shows the PWL phenolic profile of non-anthocyanin (Figure 1A) and anthocyanin (Figure 1B) analyzed by UHPLC-(ESI)-Q-TOF-MS. The total amount of phenolic compounds in the PWL was significantly higher than the one shown by 0 WL (15.59 mg/g and 24.50 mg/g, respectively) (Figure 2A). This increase was observed in all phenolic families following this order: anthocyanins > flavonols > flavanols > phenolic acids > stilbenes (Figure 2A).

Flavanols were the main family of phenolic compounds in the 0 WL, representing 43.1 % of total phenolic content, followed by anthocyanins (24.4 %, Figure 2B). However, in the PWL, flavanols and anthocyanins were found in the same proportion (33.6 % and 33.5 % of the total phenolic compounds, respectively; Figure 2B). Table 2 and Table 3 show the individual phenolic compounds content in both 0 WL and PWL. A total amount of 40 non-anthocyanins and 40 anthocyanins were identified by UHPLC-ESI-Q-TOF-MS, respectively. The major components in PWL were gallic acid (3.5 mg/g), catechin (3.3 mg/g), malvidin-3-glucoside (3.3 mg/g), procyanidin dimers (2.6 mg/g), quercetin (2.0 mg/g), malvidin-(6-acetyl)-3-glucoside (1.5 mg/g), and epicatechin (1.2 mg/g). Protein hydrolysis released large amounts of anthocyanins, especially delphinidin-3-glucoside, petunidin-3-glucoside, malvidin-(6-acetyl)-3-glucoside, malvidin-(6-coumaroyl)-3-glucoside, and malvidin-3-glucoside. Within the family of flavanols, catechin, epicatechin, and procyanidin dimer B2 were those that showed the greatest increase after hydrolysis. All the identified compounds of the flavonol family suffered an increase between 97.7–152.1% in their content after hydrolysis (Table 2), quercetin being the major flavonol. Regarding phenolic acids, the main increase after the hydrolysis was observed in p-coumaric acid. While, resveratrol and its derivatives were the stilbenes that presented the highest release after hydrolysis of WL.

### 3.3. Antioxidant and ACEi Activities of the Wine Lees

**Figure 3:**
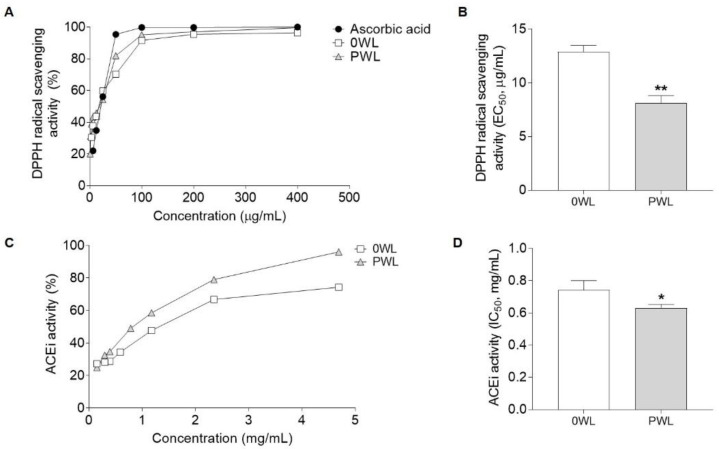
Dose–response curves of DPPH radical scavenging activity (%, **A**) and angiotensin-converting enzyme inhibitory (ACEi) activity (%, **C**) for control wine lees (0 WL) and phenolic-enriched wine lees (PWL). (**B**) shows DPPH radical scavenging activity as EC_50_ and (**D**) show ACEi activity as IC_50_. Values are the average of three replicates ± SD. Statistical differences by Student’s T-test between 0 WL and PWL are indicated (*) when *p* < 0.05 and (**) when *p* < 0.01.

Figure 3A shows an example of a dose–response curve of both wine lees used to determine their antioxidant activity as DPPH radical scavenging activity (%). EC_50_ values were 12.89 ± 0.72 µg/mL and 8.14 ± 0.81 µg/mL in the 0 WL and PWL, respectively (Figure 3B). ACEi activity of the samples was also evaluated. The PWL showed a higher ACEi activity than the 0 WL (53.6 and 35.7 %, respectively at 0.59 mg/mL) (Figure 3C). According to these percentages, the IC_50_ value was lower in the hydrolysate (0.63 ± 0.02 mg/mL) in comparison with the control 0 WL (0.74 ± 0.06 mg/mL) (Figure 3D).

### 3.4. Antihypertensive Activity of the Wine Lees

The antihypertensive effect of both 0 WL and PWL was evaluated in SHR at 5 mL/kg bw. This dose is equivalent to the doses of 18.50 and 20.01 mg GAE/kg bw and 1.95 and 3.06 mg/kg bw of phenolic compounds determined by UHPLC-(ESI)-Q-TOF-MS for 0 WL and PWL, respectively.

**Figure 4:**
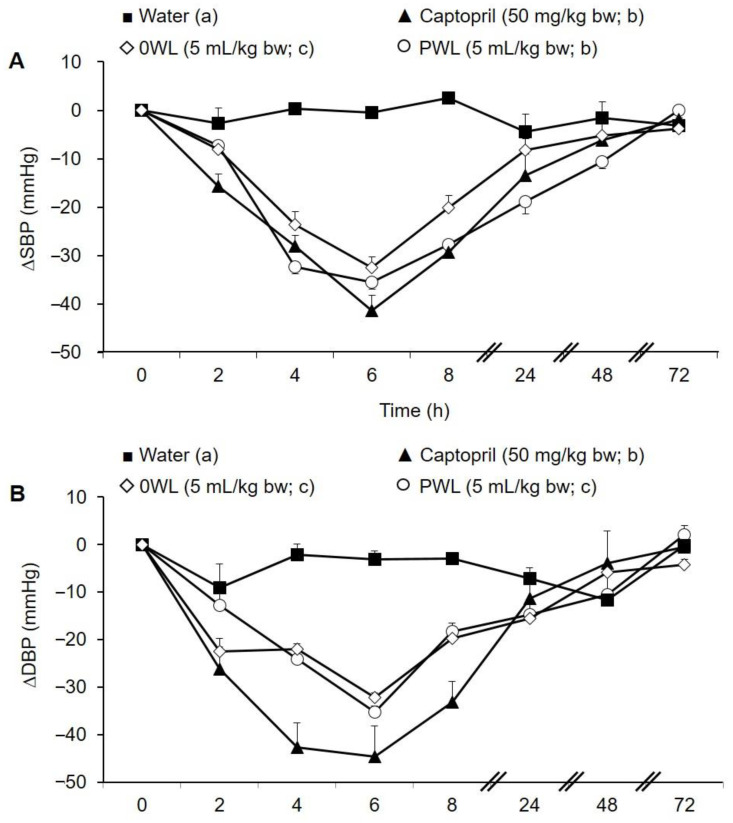
Changes in systolic (**A**) and diastolic (**B**) blood pressure (SBP and DBP, respectively) in spontaneously hypertensive rats after the administration of the following: water (■), Captopril (50 mg/kg bw; ▲), control wine lees (5 mL/kg bw; 0 WL ◊), and phenolic-enriched wine lees (5 mL/kg bw; PWL ○). Data are expressed as the mean ± SEM (n = 6). Different letters in the legend represent significant differences (*p* < 0.05). *p* was estimated by two-way ANOVA.

Water and Captopril (50 mg/kg bw) were used as negative and positive controls, respectively. Prior to oral treatment administration, animals presented SBP and DBP values characteristic of the hypertensive condition (205.6 ± 3.6 mmHg and 164.7 ± 10.0 mmHg, respectively). As shown in Figure 4, the administration of water to animals did not significantly change either SBP or DBP during the experiment. However, Captopril produced a decrease in SBP in SHR after 2 h of its administration, its maximum being at 6 h post-administration (−41.3 ± 3.2 mm Hg) (Figure 4A). The same pattern of SBP drop occurred after administration of 0 WL. A maximum SBP reduction of −32.5 ± 2.3 mmHg was observed at 6 h post-administration. Regarding the effect of the PWL, the maximum SBP decrease was observed at 4–6 h post-administration (−32.3 ± 1.5 and −35.6 ± mmHg, respectively). Notably, the BP lowering effect of PWL was greater than the antihypertensive effect shown by 0 WL. Interestingly, no significant differences were found between the antihypertensive effect of Captopril and PWL (two-way ANOVA). In addition, the duration of the effect was different. The antihypertensive effect produced by PWL was more prolonged than the one showed by 0 WL or Captopril. Basal SBP levels were recovered at 24, 48, and 72 h post-administration for 0 WL, Captopril and PWL, respectively.

Regarding DBP values, Captopril administration also produced a significant reduction, reaching a maximum decrease 4 h post-administration (−42.7 ± 5.2 mmHg) (Figure 4B). The antihypertensive effect was observed up to 24 h after Captopril administration. 0 WL and the hydrolysate also produced a significant antihypertensive effect from 2 h post-administration, reaching the maximum DBP values at 6 h (−32.2 ± 5.2 and −35.2 ± 1.2 mmHg, respectively). No differences in DBP were found between the two WL samples under study.

## 4. Discussion

Winery by-products such as seeds, pomace or skin, have shown to be rich in phenolic compounds [41]. In fact, these by-products of the winemaking process have been used to obtain extracts, rich in phenolic compounds with beneficial properties such as antioxidant or antihypertensive activities [9,10,11,19,21]. However, some of the phenolic compounds are located inside the cells (and in some cases bound to different compounds such as proteins) making it difficult to remove them from the food matrix and therefore decreasing their full potential [42]. Recently, our group demonstrated the antihypertensive effect of a soluble fraction of WL from Cabernet variety after its acute administration (5 mL/kg bw) to SHR [19]. This effect was attributed to their high content of phenolic compounds. Nevertheless, this fraction did not contain the non-soluble phenolic compounds since no method of extraction was applied.

Different methodologies have been described to facilitate the release of phenolic compounds from the matrix [43]. Enzyme-assisted extraction has emerged as a more eco-friendly alternative to conventional solvent-based extraction methods [24]. In addition, enzymatic hydrolysis is a more efficient extraction method that increases the extractability of phenolic compounds from the food matrix (including those non-extractable as proanthocyanidins) using neither organic solvents nor any other toxic chemicals. Moreover, it is a cost-efficient method to convert by-products into new and safe food ingredients or products with enhanced nutritional value and functionality [25]. This technique is based on the use of enzymes to break down different components of the cell to enhance the release of phenolic compounds [26].

Therefore, since WL with a higher content in phenolic compounds could potentially exhibit a stronger functionality, enzyme-assisted extraction was used to release the phenolic compounds present in the non-soluble fraction of WL. The enzymatic preparation Flavourzyme^®^ was used because it is widely used for protein hydrolysis in industrial and research applications [44]. To the best of our knowledge, it is the first time that this method has been used in WL to extract phenolic compounds. The results showed changes in the amino acid composition of PWL with respect to 0 WL, indicating that protein hydrolysis occurred. This protein hydrolysis was expected since Flavourzyme^®^ is an enzymatic preparation with peptidase activity. The amino acid residues Pro, Ile, Leu, Val, and Trp were those that increased their content in the WL hydrolysate (PWL) more than the 0 WL control. In addition, the hydrolysis of WL also produced an increase in the phenol content quantified by the Folin–Ciocalteu method and by UHPLC-(ESI-)-Q-TOF-MS, which increased 57.20%.

The specific locations of phenolic compounds, their type of bonding and possible physical entrapment in WL is largely unknown at present. Methods such as ultrasound [45,46] and microwave [47] assisted extraction have been used to enhance the extraction of active compounds from many vegetable matrixes, including WL. In this study, enzyme-assisted extraction using Flavourzyme^®^ was demonstrated to be a useful technique to release phenolic compounds from WL. In concordance with these results, Senevirathne et al. showed that the hydrolysis of blueberries, with different enzymes, including the enzymatic preparation Flavourzyme^®^, released phenolic compounds [48]. In the process of wine maceration, the phenolic compounds from grapes are transferred to wine. However, a high proportion of these compounds remains in winery by-products such as WL. The presence of phenolic compounds in WL is due to the great adsorption capacity of the yeast cell wall used in the winemaking process [49]. The phenolic profile present in the WL depends on the type of grapes and other factors involved in the winemaking [50]. The WL used in this study were obtained from grapes of the variety Cabernet, and their functionality was linked to the high amount of anthocyanins and flavanols present in these lees [19]. In this study, the hydrolysis of WL caused an increase in content of all the phenolic compound families. However, anthocyanins and flavonols were the categories of polyphenols whose concentration increased more in the PWL compared to WL, doubling both their content. Regarding the phenolic profile, PWL were especially rich in flavanols (33.56%) and anthocyanin (33.52%), the flavanols being catechin and epicatechin and the anthocyanins delphinidin-3-glucoside, petunidin-3-glucoside, and malvidin-(6-acetyl)-3-glucoside, the most increased by the hydrolysis process. Thus, the application of this enzymatic-assisted extraction method to WL produced PWL rich in gallic acid, catechin, malvidin-3-glucoside, procyanidin dimers, quercetin, malvidin-(6-acetyl)-3-glucoside, and epicatechin.

Phenolic compounds exhibit numerous beneficial effects. Among them, their radical scavenging capacity stands out, giving them antioxidant properties [5]. Thus, since the hydrolysis causes a release of phenolic compounds, PWL should show a higher antioxidant capacity than 0 WL. The results in this study showed that both samples presented antioxidant activity although, as expected, the hydrolysate presented a more potent antioxidant capacity. This improvement in the antioxidant effect could be linked to the higher content of anthocyanins, since a good correlation between anthocyanin compounds and antioxidant activity has been reported for different WL extracts [51]. Both DPPH radical scavenging activities are higher than previously reported for other winery by-products, i.e., a grape stem phenolic extract [20,52]. Along with other beneficial effects, the antioxidant properties of phenolic compounds have also been related to the improvement of HTN [53,54]. In fact, one of the underlying mechanisms involved in the endothelial damage is oxidative stress, which increases the contractibility of the vascular smooth muscle and promotes its proliferation [55]. Furthermore, free radicals in the endothelium can scavenge nitric oxide (NO), avoiding NO-dependent vasodilation and stimulating the production of pro-inflammatory agents and endothelium-derived vasoconstrictor factors [56].

The RAAS is another key factor in the maintenance of arterial BP. One of the main components of this system is the ACE. In fact, the inhibition of this enzyme is usually used to select antihypertensive compounds. ACE inhibitors such as Enalapril or Captopril are usually used as treatment against HTN [57]. ACE catalyzes the conversion of angiotensin I into the potent vasoconstrictor angiotensin II [58]. The ACEi properties of the soluble fraction of the WL have been previously reported [19]. Since the ACEi activity of phenolic compounds has also been reported [17], the hydrolysate PWL, as expected, exerted a more potent ACEi activity than its control counterpart 0 WL.

Subsequently, the antihypertensive effect of the hydrolysate was evaluated *in vivo*, using SHR as a hypertensive animal model. This is the one well-established of the most used experimental models of HTN, very similar to the HTN found in humans [59]. The administration of 5 mL/kg bw of the hydrolysate produced a potent antihypertensive effect, reaching the maximum SBP decrease between 4 and 8 h post-administration (−32.29 ± 1.46 mmHg). In addition, this effect was long lasting, remaining until 48 h post administration. A similar time response of the SBP-lowering effect has been shown by hydrolysates obtained from other food by-products such as garlic protein hydrolysates, which exhibited the maximum effect between 4–6 h post-administration [60]. It is noteworthy that, since it lasted longer, the antihypertensive effect of the PWL was more potent than the one shown by the commercially available drug Captopril or the 0 WL. Considering that HTN is a chronic pathology that needs lifetime treatment, the use of strategies with long lasting antihypertensive effects is always desired. Therefore, the antihypertensive properties of PWL seem more favorable.

It is known that phenolic compounds have numerous effects on health, including antihypertensive effects [61]. The improvement of the antihypertensive effect of the WL using enzymatic hydrolysis is linked to the release of phenolic compounds. In this sense, intake of compounds rich in anthocyanins has shown to be associated with a lower arterial stiffness and with a reduction in BP [62,63,64]. More specifically, malvidin-3-glucoside, present in high amounts in the PWL, has shown to be a potent vasodilator [65]. Furthermore, compounds rich in flavanols such as catechin, epicatechin or procyanidins, have been shown to have antihypertensive effect in rats and humans [9,66,67]. In our previous study, we also related the WL bioactivity to the content of flavanols and anthocyanins [19], which is in line with the present results. Moreover, PWL showed an increment in flavonols, especially in quercetin. It is known that flavonols, such as quercetin, improve vascular endothelial function and reduce BP [68,69]. In this regard, several studies have reported BP- lowering effects of quercetin in several animal models of HTN (10 mg/kg/day for 4 or 5 weeks) and in hypertensive patients (730 mg/day for 28 days) [70,71]. In addition, PWL also presented high levels of gallic acid. Kang et al. showed that the administration of 40 mg/kg bw of this phenolic acid to SHR produced an antihypertensive effect similar to the observed by Captopril [72].

Taken together, the data obtained in this study show that the hydrolysis of WL is a good strategy to release phenolic compounds, specifically anthocyanins and flavonols, and the results strongly suggest that these phenolic compounds enhance the antihypertensive effect of WL. Moreover, taking into account that WL also contain ethanol, which can reach values similar to wine of at least 8.5% [73], an improvement of WL antihypertensive activity using dealcoholized WL might be attained.

## 5. Conclusions

The phenolic extraction from the solid fraction of WL via enzymatic hydrolysis is a useful method to obtain phenolic-enriched WL with enhanced antioxidant, ACEi and antihypertensive properties. As a result, a WL fraction rich in flavanols, anthocyanins, phenolic acids, and flavonols was obtained. The phenolic composition is considered of special relevance since multiple beneficial effects have been linked to these phenolic families, including antihypertensive properties. Therefore, this study opens the door to the wine industry for the commercial use of PWL due to its high content of phenolic compounds. Moreover, the enzyme-assisted extraction of phenolic compounds also modified the amino acid content of WL, indicating that protein hydrolysis was taking place. Therefore, the release of other molecules with antihypertensive properties, different to phenolic compounds, such as bioactive peptides should not be ruled out. Moreover, as in addition to phenolic compounds, WL contains ethanol, it would be of interest to investigate the BP- lowering effect of the dealcoholized WL samples. Finally, further studies would be needed to confirm the long-term effect of PWL in hypertensive and normotensive rats.

## 6. Patents

Patent application “Wine lees, derivatives thereof and their uses”: application number EP20382358.8 and and PCT/EP2021/053051.

## Figures and Tables

**Table 1:** Total amino acid content of 0 WL and PWL by UHPLC-Q-TOF/MS.

Amino Acids	0 WL (µg/g)	PWL (µg/g)
Alanine	706.3 ± 62.8	854.5 ± 70.1
Arginine	110.5 ± 12.5	195.2 ± 19.9
Asparagine	273.6 ± 59.5	417.6 ± 91.0
Aspartate	307.9 ± 37.4	702.9 ± 78.3
Glutamate	1086.1 ± 265.5	1278.4 ± 325.8
Glutamine	N.D	N.D
Glycine	345.5 ± 65.2	442.1 ± 68.8
Histidine	75.6 ± 9.3	135.4 ± 7.2
Isoleucine	358.8 ± 54.7	2014.6 ± 256.2 **
Leucine	435.8 ± 74.4	2857.6 ± 445.7 **
Lysine	143.7 ± 28.2	238.4 ± 46.6
Phenylalanine	64.9 ± 11.7	189.9 ± 34.8
Proline	19,828.7 ± 1230.8	28,623.8 ± 1761.1 **
Serine	517.0 ± 188.6	1091.9 ± 348.0
Threonine	326.2 ± 87.7	720.5 ± 181.0
Tryptohan	N.D	52.9 ± 18.6 *
Tyrosine	46.8 ± 7.2	152.8 ± 19.9
Valine	181.7 ± 29.4	1187.0 ± 172.4 *
Methionine	25.0 ± 7.6	46.8 ± 13.1
Cystine	N.D	N.D
Hydroxiproline	N.D	N.D
TOTAL	24,834.1	41,202.3

N.D: no detected. Data are expressed as mean (µg/g of dry sample) ± SD. Statistical differences by Student’s T-test between control wine lees (0 WL) and phenolic-enriched wine lees (PWL) are indicated as (*) when *p* < 0.05 and (**) when *p* < 0.01.

**Table 2:** Non-anthocyanin characterization by UHPLC-(ESI-)-Q-TOF-MS in 0 WL and PWL.

	Compound	R.T. (min)	[M-H]-	Fragment (*m/z*)	0 WL (µg/g)	PWL (µg/g)
	Flavanols					
1	Catechin	8.17	289.0718		2681.20 ± 19.20	3289.60 ± 20.80 *
2	Catechin gallate ^a^	6.66	441.0827	289.07209	18.00 ± 0.40	16.40 ± 0.40 **
3	Epicatechin	9.96	289.0718		1035.60 ± 6.00	1242.00 ± 6.80 *
4	(Epi)catechin *O*-glucoside iso1 ^b^	6.55	451.1246	289.0721	20.80 ± 0.00	22.80 ± 0.00 *
5	(Epi)catechin *O*-glucoside iso2 ^b^	7.41	451.1246	289.0721	10.80 ± 0.00	16.40 ± 0.00 *
6	(Epi)catechin *O*-glucoside iso3 ^b^	8.37	451.1246	289.0721	62.80 ± 1.20	70.80 ± 1.20 *
7	Procyanidin dimer B2	9.30	577.1387	289.0733	514.40 ± 0.40	634.00 ± 0.40 **
8	Procyanidin dimer iso1 ^c^	7.68	577.1387	289.0733	1020.80 ± 6.00	1178.00 ± 6.40 *
9	Procyanidin dimer iso2 ^c^	7.97	577.1387	289.0733	276.80 ± 2.00	334.40 ± 2.00 *
10	Procyanidin dimer iso3 ^c^	8.18	577.1387	289.0733	85.20 ± 0.80	86.80 ± 0.40
11	Procyanidin dimer iso4 ^c^	8.99	577.1387	289.0733	211.20 ± 0.00	262.80 ± 0.00 *
12	Procyanidin dimer iso5 ^c^	11.14	577.1387	289.0733	70.40 ± 0.80	106.00 ± 1.20
13	Procyanidin trimer iso1 ^c^	5.46	865.2016	577.1369	206.00 ± 4.00	251.60 ± 4.40
14	Procyanidin trimer iso2 ^c^	8.67	865.2016	577.1369	184.00 ± 10.40	274.00 ± 13.60 *
15	Procyanidin trimer iso3 ^c^	8.89	865.2016	577.1369	72.40 ± 0.40	84.00 ± 0.40 *
16	Procyanidin trimer iso4 ^c^	10.55	865.2016	577.1369	68.80 ± 3.60	89.60 ± 0.40 *
17	Procyanidin trimer iso5 ^c^	10.71	865.2016	577.1369	177.60 ± 3.60	265.60 ± 4.40 *
	Flavonols					
18	Quercetin	17.80	301.0372		888.40 ± 4.80	1954.40 ± 9.20 **
19	Quercetin-3-*O*-glucoside ^d^	13.00	463.0904	301.0361	19.20 ± 0.40	48.40 ± 1.20 **
20	Quercetin-3-*O*-glucuronide ^d^	12.95	477.0702	301.0369	115.20 ± 0.80	255.20 ± 1.20 **
21	Kaempferol ^d^	20.07	285.0405		319.60 ± 1.60	632.00 ± 2.40 **
22	Kaempferol-3-*O*-glucuronide ^d^	14.22	461.0763	285.0412	27.60 ± 0.40	66.00 ± 0.80 **
23	Isorhamnetin ^d^	20.31	315.0531		203.20 ± 2.40	481.60 ± 5.20 **
	Phenolic acids					
24	Gallic acid	3.13	169.0193		2734.80 ± 93.60	3496.80 ± 106.40
25	Caffeic acid	8.63	179.0401		70.80 ± 0.80	97.20 ± 1.20 *
26	Caffeic acid *O*-glucoside iso1 ^e^	7.64	341.0878	179.0350	22.40 ± 0.80	23.20 ± 0.80
27	Caffeic acid *O*-glucoside iso2 ^e^	8.29	341.0878	179.0350	22.40 ± 1.20	19.60 ± 0.80
28	p-Coumaric acid	10.65	163.0439		28.00 ± 0.40	117.60 ± 1.20 **
29	4-Hydroxybenzoic acid	8.17	137.0243		58.40 ± 2.00	66.40 ± 2.00
30	Ferulic acid	12.00	193.0506		13.20 ± 0.40	18.80 ± 0.40 *
31	Vanillic acid	8.51	167.0350		76.40 ± 2.40	90.40 ± 2.80
	Stilbenes					
32	trans-Resveratrol ^f^	15.73	227.0714		120.40 ± 0.80	164.00 ± 0.80 **
33	Resveratrol iso1 ^f^	18.00	227.0714		66.00 ± 0.40	152.40 ± 0.80 **
34	Resveratrol *O*-glucoside iso1 ^f^	12.44	389.1242	227.0721	30.00 ± 0.40	56.40 ± 0.80 **
35	Resveratrol *O*-glucoside iso2 ^f^	14.92	389.1242	227.0721	88.00 ± 1.60	138.00 ± 2.40 **
36	Piceatannol ^f^	2.59	243.0663	203.0727	124.40 ± 2.00	136.80 ± 1.60 *
37	Piceatannol 3-*O*-glucoside iso1 ^f^	12.89	405.1208	243.0670	5.60 ± 0.00	9.60 ± 0.40 *
38	Piceatannol 3-*O*-glucoside iso2 ^f^	13.15	405.1208	243.0670	2.40 ± 0.00	4.80 ± 0.00 *
39	Viniferin-iso1 ^f^	19.53	453.1344	116.9291	4.40 ± 0.00	6.40 ± 0.00 *
40	Viniferin-iso2 ^f^	19.92	453.1344	116.9291	20.80 ± 0.40	29.20 ± 0.80 *

Abbreviations: Retention time (R.T.); Control WL (0 WL); Phenolic-enriched wine lees (PWL). ^a^ Quantified using catechin calibration curve. ^b^ Quantified using epicatechin calibration curve. ^c^ Quantified using procyanidin dimer B2 calibration curve. ^d^ Quantified using quercetin calibration curve. ^e^ Quantified the caffeic acid calibration curve. ^f^ Quantified using resveratrol calibration curve. Statistical differences by Student’s *t*-test between 0 WL and PWL are indicated (*) when *p* < 0.05 and (**) when *p* < 0.01.

**Table 3:** Anthocyanin characterization by UHPLC-(ESI+)-Q-TOF-MS in 0 WL and PWL.

	Anthocyanins	R.T. (min)	[M-H]+	Fragment (*m/z*)	0 WL (µg/g)	PWL (µg/g)
1	Gallocatechin-malvidin-3-glucoside dimer ^a^	3.58	797.2035		1.60 ± 0.04	4.46 ± 0.11 *
2	Malvidin-3-glucoside-(epi)catechin ^a^	4.84	781.1974		16.67 ± 0.13	19.27 ± 0.14 *
3	Delphinidin-3-glucoside ^b^	5.06	465.1028	303.0511	184.88 ± 1.82	573.34 ± 5.64 **
4	Cyanidin-3-glucoside ^b^	5.85	449.1078	287.0531	21.72 ± 0.81	48.11 ± 1.79 *
5	Petunidin-3-glucoside ^c^	6.47	479.1184	317.0669	195.11 ± 2.26	513.73 ± 5.96 **
6	Petunidin-3-glucoside-pyruvic acid ^c^	7.05	547.1082	385.0547	1.61 ± 0.04	3.54 ± 0.09 *
7	Peonidin-3-glucoside ^c^	7.14	463.1235	301.0717	151.78 ± 2.50	312.22 ± 5.14 *
8	Malvidin-3-glucoside ^a^	7.48	493.1341	331.0843	1780.76 ± 20.01	3334.75 ± 37.47 *
9	Peonidin-3-glucoside-pyruvic acid ^c^	7.81	531.1133	369.0607	0.93 ± 0.03	1.90 ± 0.06 *
10	Delphinidin-(6-acetyl)-3-glucoside ^b^	7.87	507.1133	303.0496	47.82 ± 0.90	151.91 ± 2.85 *
11	Visitin A (malvidin-3-glucoside-pyruvic acid) ^a^	8.11	561.1239	399.0730	14.80 ± 0.13	31.02 ± 0.27 **
12	Visitin B (malvidin-3-glucoside-acetaldehyde) ^a^	8.32	517.1341	355.0826	24.08 ± 0.59	70.03 ± 1.73 *
13	Malvidin-3-glucoside-ethyl-(epi)catechin ^a^	8.40	809.2287		4.62 ± 0.03	8.91 ± 0.05 *
14	Cyanidin-(6-acetyl)-3-glucoside ^b^	8.45	491.1184	491.1189	15.21 ± 0.23	33.29 ± 0.50 *
15	Acetylvisitin A ^a^	8.50	603.1344	399.0718	13.66 ± 0.44	19.65 ± 0.63 *
16	Malvidin-3-glucoside-ethyl-(epi)catechin ^a^	8.57	809.2287		17.54 ± 0.24	31.82 ± 0.43 *
17	Petunidin-(6-acetyl)-3-glucoside ^c^	8.66	521.1378	317.0667	55.81 ± 1.87	150.18 ± 5.04 **
18	Malvidin-3-glucoside-ethyl-(epi)catechin ^a^	8.75	809.2287		22.92 ± 0.73	42.10 ± 1.34 **
19	Acetylvisitin B ^a^	8.77	559.1446	355.0813	14.50 ± 0.44	36.86 ± 1.12 *
20	Peonidin-(6-acetyl)-3-glucoside ^c^	9.08	505.1341	301.0714	55.47 ± 1.24	124.75 ± 2.80 *
21	Delphinidin-(6-coumaroyl)-3-glucoside ^b^	9.08	611.1395	303.0508	14.72 ± 0.27	48.48 ± 0.89 *
22	Malvidin-(6-acetyl)-3-glucoside ^a^	9.13	535.1446	331.0836	727.35 ± 0.84	1503.53 ± 1.74 **
23	Coumaroylvisitin A ^a^	9.29	707.1607	399.0718	2.88 ± 0.07	6.18 ± 0.15 *
24	Malvidin-(6-caffeoyl)-3-glucoside ^a^	9.41	655.1657	331.0808	5.99 ± 0.27	13.98 ± 0.63 *
25	Cyanidin-(6-coumaroyl)-3-glucoside ^b^	9.42	595.1446	287.0560	5.01 ± 0.16	13.77 ± 0.43 *
26	Catechin-ethyl-malvidin-3-acetylglucoside dimer ^a^	9.43	851.2511		10.30 ± 0.31	19.05 ± 0.56 **
27	Petunidin-(6-coumaroyl)-3-glucoside ^c^	9.52	625.1552	317.0662	21.11 ± 0.36	63.19 ± 1.08 *
28	Pinotin A (malvidin-3-glucoside-vinylcatechol) ^a^	9.53	625.1552	463.0998	23.80 ± 0.51	71.25 ± 1.52 *
29	Malvidin-glucoside-vinyl-catechin ^a^	9.56	805.1974		1.46 ± 0.03	4.23 ± 0.09 *
30	Coumaroylvisitin B ^a^	9.58	663.1708	355.0822	8.53 ± 0.28	23.20 ± 0.76 *
31	Malvidin-3-glucoside-vinylguaiacol ^a^	9.63	639.1708	331.0823	10.07 ± 0.20	27.49 ± 0.54 *
32	Catechin-ethyl-malvidin-3-coumaroylglucoside dimer ^a^	9.70	955.2785		5.95 ± 0.11	13.77 ± 0.25 *
33	Catechin-ethyl-malvidin-3-acetylglucoside dimer ^a^	9.81	851.2511		1.93 ± 0.06	4.27 ± 0.14 *
34	Peonidin-(6-coumaroyl)-3-glucoside ^c^	9.87	609.1603	301.0716	38.72 ± 1.08	85.15 ± 2.37 *
35	Malvidin-(6-coumaroyl)-3-glucoside ^a^	9.92	639.1708	331.0823	274.63 ± 0.60	768.26 ± 1.69 **
36	Malvidin-glucoside-vinyl-catechin ^a^	9.99	805.1974		1.58 ± 0.02	3.74 ± 0.05 **
37	Acetyl-pinotin A ^a^	10.19	667.1657		0.08 ± 0.00	0.26 ± 0.00 *
38	Malvidin 3-*O*-glucoside 4-vinylphenol (Pigment A) ^a^	10.22	609.1603	447.1079	7.40 ± 0.08	19.53 ± 0.21 **
39	Catechin-ethyl-malvidin-3-coumaroylglucoside dimer ^a^	10.33	955.2785		1.14 ± 0.01	3.06 ± 0.02 *
40	Malvidin acetyl 3-*O*-glucoside 4-vinylphenol (Acetyl-pigment A) ^a^	10.50	651.1708	447.1076	4.46 ± 0.17	10.42 ± 0.39 *

Abbreviations: Retention time (R.T.); Control WL (0 WL); Phenolic-enriched wine lees (PWL). ^a^ Quantified using calibration curve of malvidin-3-*O*-glucoside. ^b^ Quantified using calibration curve of cyanidin-3-*O*-rutinoside. ^c^ Quantified using calibration curve of peonidin-3-*O*-rutinoside. Statistical differences by Student *t*-test between 0 WLE and PWL are indicated (*) when *p* < 0.05 and (**) when *p* < 0.01.

## Data Availability

Not applicable.
